# Quality of Life in Hematologic Malignancy in the Eastern Mediterranean Region: A Systematic Review

**DOI:** 10.7759/cureus.32436

**Published:** 2022-12-12

**Authors:** Marwa M Alhamss, Lein F Mathbout, Rama B Nassri, Mohamad S Alabdaljabar, Shahrukh Hashmi, Ibrahim N Muhsen

**Affiliations:** 1 College of Medicine, Alfaisal University, Riyadh, SAU; 2 Department of Internal Medicine, Mayo Clinic, Rochester, USA; 3 Division of Hematology/Oncology/Blood and Marrow Transplant, Mayo Clinic, Rochester, USA; 4 Department of Hematology and Medical Oncology, Baylor College of Medicine, Houston, USA

**Keywords:** cancer survival, cancer patients, hematological malignancies, eastern mediterranean region, quality of life (qol)

## Abstract

Health-related quality of life (HRQoL) indicates patients’ overall health and is an essential aspect of cancer care. Although multiple studies have addressed the various aspects of HRQoL in cancer patients, few studies have investigated HRQoL in hematologic malignancy patients in the Eastern Mediterranean region (EMR). This review conducted an electronic search using OVID-Medline to identify HRQoL-related articles involving hematologic malignancy patients in the EMR. Eight studies met the inclusion criteria. Two studies validated translated QoL psychometric instruments, three were observational studies, and three were interventional studies. Except for the validation studies, all studies discussed HRQoL in leukemia patients. Our review highlighted a scarcity in the number of studies focusing on patients with hematological malignancies in this region. The included studies demonstrated the negative impact of hematological malignancies and therapies on patients’ HRQoL. In addition, the studies displayed the association between physical symptoms and QoL of cancer patients, necessitating the importance of addressing these symptoms. The studies were limited by publication year, the number of patients, geographical locations, and disease entities. Future studies in this area are encouraged to help understand factors affecting HRQoL in the EMR region and ways to improve it. Consequently, further research is needed to establish translated and validated QoL assessment instruments that target patients in the EMR using the most common tools including the Short-Form 36-item Health Survey and the European Organization for the Research and Treatment of Cancer Quality of Life Questionnaire.

## Introduction and background

Hematological malignancies are a major cause of mortality in adults and children, with lymphoma, leukemia, and multiple myeloma (MM) being the most common subtypes. In 2018, the estimated number of patients who were diagnosed in the United States with non-Hodgkin lymphoma (NHL) was 81,560, and 8,830 patients were diagnosed with Hodgkin lymphoma (HL) [1]. The global incidence of NHL and HL in 2020 was approximately 500,000 (2.8%) and 80,000 (0.4%), respectively [2]. Moreover, leukemia is a common malignancy, particularly in the pediatric age groups, with close to 500,000 (2.5%) newly diagnosed patients reported globally in 2018 [1,2]. MM is the least common subtype compared to the other types, with 176,000 new cases reported globally in 2020 [2].

Given the high incidence rate, improved therapeutic options, and the increased number of survivors, quality of life (QoL) has become a crucial component of the current approach to patients with hematologic malignancies. Health-related QoL (HRQoL) captures information regarding patients’ mental and physical health, indicating their overall health [3]. Multiple studies have addressed the different aspects of HRQoL in cancer patients. These studies have shown the deterioration in different QoL parameters in patients with both solid and hematologic malignancies [4,5]. However, the majority of these studies were conducted in developed countries. The Eastern Mediterranean region (EMR), which includes 22 countries located in Asia and North Africa, is collectively referred to as EMR by the World Health Organization (WHO) [6]. These countries are on two continents, with different economic structures, resources, traditions, cultures, and languages. The lack of data from EMR and the small number of formal national cancer control programs can hide different results compared to other countries, including North America, Europe, and Japan. Moreover, countries in the EMR vary considerably in many aspects, including socioeconomic factors, organization of services, level of complexity of healthcare systems, as well as cultural and language differences. Treatments such as surgery, radiation therapy, and chemotherapy are being used in better ways in addition to newer treatments such as targeted therapy and immunotherapy which result in extending and saving lives. Cancer research leads to new advances in cancer screening, treatment, and survivorship care. Here, we aim to perform a literature review focusing on the impact of hematologic cancer on HRQoL, in general, and in EMR countries, in particular.

## Review

Methodology

This study was not registered and did not receive any financial support. The search strategy was comprehensive and targeted all studies on QoL conducted among cancer patients in the EMR. The search strategy included studies limited to OVID-Medline (R) published from 1946 to November 29, 2021. The search strategy used Boolean logic, with terminology including hematological malignancies-related terms, both general (e.g., hematologic malignancy) and type-specific (i.e., leukemia, lymphoma, hematopoietic cell transplant, and MM), as well as the term “quality of life.” Terms were used as both keywords and Medical Subject Headings terminology. Additionally, all country names of the EMR were used as keywords to limit the search to the region of interest.

We included primary research articles, including interventional and observational studies with different designs, such as cross-sectional, prospective, and comparative. Studies concerning HRQoL and hematologic malignancies included primary human studies, reviews, concept papers, or case reports/series. Additionally, we included studies that assessed QoL using a validated QoL assessment tool (studies that used tools to assess only one aspect such as fatigue or sleep were excluded). International studies that included a sample from an EMR country were excluded unless specific analysis by region was performed. Moreover, studies that included a general sample of cancer patients without cancer type-specific analysis were excluded. Publications were included regardless of the publication year. Finally, all included studies were published in the English language only.

Results

**Figure 1:**
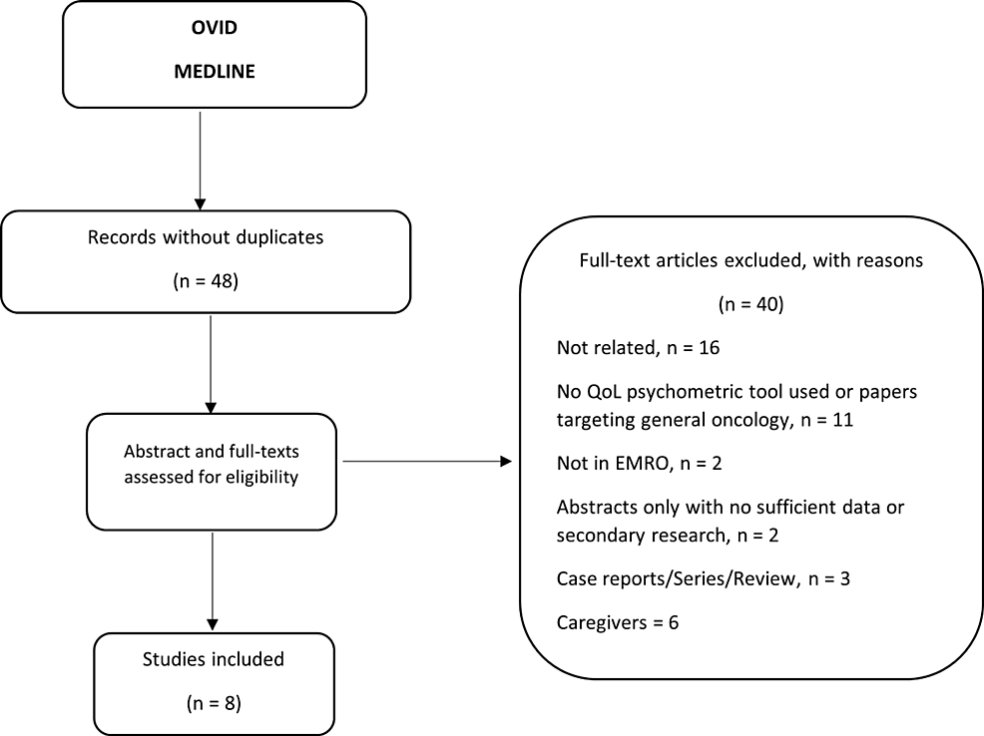
Schematic figure of the search strategy.

The initial search yielded 48 studies that were screened for eligibility. Out of the 48 studies, 40 were excluded because they did not meet the selection criteria (Figure 1). Only eight studies met the inclusion criteria, including two studies that used a hematologic malignancy population to validate a QoL translated psychometric instrument, three observational studies, and three interventional studies. The two validation studies included MM and lymphoma patients, while all interventional and observational studies focused on QoL in leukemia patients.

In certain instances, multiple studies discussed the general oncology population including hematologic malignancy patients [7,8]. These studies provided helpful insights but were not specific to hematologic malignancy patients. For instance, Mosleh [7] reported predictors for QoL in 26 lymphoma patients and found the following factors to be associated with an improved QoL: higher educational level, fewer hospital admissions, and low anxiety and depression scores. Additionally, Ahmed et al. [9] showed that patients who did not exercise and had a new cancer diagnosis were associated with lower QoL in a study sample of 438 Saudi cancer patients. Leukemia patients had lower QoL scores than lymphoma and other solid malignancy patients. Lymphoma patients had higher global QoL scores compared to other cancer types.

Quality of Life Measurement and Validation Studies

Several QoL measuring tools are available, with the most common being the Short-Form 36-Item Health Survey (SF-36) and the European Organization for Research and Treatment of Cancer quality of life (QLQ-C30). QLQ-C30 was developed by a European organization for the research and treatment of cancer and consists of 30 items divided between functional and symptomatic scales. The functional scales include the general health condition and physical, emotional, cognitive, social, and psychological function, while the symptomatic scales assess fatigue, pain, nausea/vomiting, dyspnea, insomnia, loss of appetite, constipation, and diarrhea [10,11]. Both the SF-36 and QLQ-C30 were frequently utilized in the studies included in this review. The language barrier is a significant challenge in ensuring that these tools are accurate. Several studies have translated the content and experimented with the translated questionnaires on pilot samples such as Arabic and Persian (widely spoken Eastern Mediterranean languages) [12,13].

Two studies were conducted specifically on hematologic malignancy patients to translate and validate the tools in the EMR. Soudy et al. [14] translated the Functional Assessment of Cancer Therapy-Bone Marrow Transplantation (FACT-BMT) for hematopoietic cell transplant patients. FACT-BMT is a brief self-administered questionnaire that was created in the English language in 1997 [15]. The study included 108 Saudi patients with relapsed and refractory lymphoma who underwent chemotherapy and autologous hematopoietic cell transplantation. The study validated the translation (Cronbach’s α = 0.9), which was approved by FACIT.org [16]. The study also showed that patients had normal QoL. Conversely, Ahmadzadeh et al. [17] translated the QLQ-MY20 (myeloma module) into the Persian language. The sample included 215 Iranian MM patients. The survey was also validated (Cronbach’s α = >0.80).

Observational Studies

Three observational studies met our inclusion criteria (Table 1). All studies were conducted among leukemia patients [18-20]. The three studies included 572 patients, with 338 acute myeloid leukemia (AML) patients and 234 acute lymphocytic leukemia (ALL) patients. Two studies included both ALL and AML adult patients, and one study included pediatric ALL patients. Two out of the three studies were retrospective and cross-sectional in design [18], while the third was a prospective study [19]. Two studies were done in Iran, while only one study was done in Egypt. Studies used different psychometric instruments, including SF-36 [20], QLQ-C30 [19], and Physical, Cognitive, Affective, Social, Economic, and Ego functioning (PCASSE) [18].

**Table 1:** Quality of life observational studies in the Eastern Mediterranean region. AML = acute myeloid leukemia; ALL = acute lymphocytic leukemia; QoL = quality of life; SF-36 = Short-Form 36-Item Health Survey; AYA = adult and young adults; QLQ-C30 = the European Organization for Research and Treatment of Cancer quality of life; PCASEE = Physical, Cognitive, Affective, Social, Economic, and Ego Functioning

Authors (country of publication)	Population (% of males)	Disease status/Treatment received	Mean age	Psychometric instruments used	Major findings
Miladinia et al., 2017 [20] (Iran)	406 adult acute leukemia patients: 308 AML and 98 ALL (54.2) patients	Newly diagnosed and receiving chemotherapy	33	Iranian SF-36	There was a statistically significant association between pain, fatigue, sleep disorders, and QoL. Pain, fatigue, and sleep disorders had the predictive power for QoL, with pain being the strongest predictor. Men were found to have lower QoL when compared to females
Malihi et al., 2013 [19] (Iran)	63 AYA and adult acute leukemia patients: 33 ALL and 30 AML (65.1) patients	Patients undergoing induction chemotherapy	33	QLQ-C30, version 3	Induction chemotherapy resulted in a significant decrease in global QoL and multiple dimensions of QoL
Khalifa et al., 2014 [18] (Egypt)	103 children with ALL	Patients at different stages of the disease, including diagnosis, initial remission, active treatment, survival, and relapsing	10	PCASEE	Psychiatric morbidity was evident in nearly 60% of leukemic children and their parents. There was an increased risk of cognitive impairment

The studies illustrated the significant impact of hematological malignancies on the overall QoL and HRQoL [18-20]. The largest study included more than 400 patients with acute leukemia [20] and investigated the relationship between fatigue, pain, sleep, and QoL. Fatigue, pain, and sleep disorders were common in acute leukemia adult patients. In this study, the SF-36 was used, which showed decreased QoL in patients with acute leukemia in their first year since diagnosis. Moreover, the study showed that fatigue, pain, and sleep were predictors of decreased QoL, with pain being the strongest predictor. Males were found to have lower QoL when compared to females, particularly in the dimensions of physical function and role, bodily pain, and general and mental health. The type of acute leukemia was not a predictor of global QoL. For instance, Malihi et al. [19] demonstrated that adults and adult and young adult Iranians with acute leukemia were at risk of worsening nutritional status and global HRQoL after induction chemotherapy. The common side effects before and after chemotherapy included appetite loss, nausea, and dry mouth. Using the QLQ-C30, the authors found that induction chemotherapy resulted in a significant decrease in QoL in patients, particularly in the social, emotional, and physical functions, without having a significant effect on cognitive function. The two studies illustrated the importance of addressing patients’ symptoms given the direct effect on their QoL.

On the other hand, Khalifa et al. [18] studied the psychiatric effect of ALL on pediatric patients. Over 100 patients were included at various stages of the disease (initial diagnosis to relapse) with the PCASEE questionnaire used to evaluate QoL. The study found a high reported psychiatric morbidity in 60% of patients after undergoing testing that included rating different psychiatric conditions such as anxiety, depression, and post-traumatic stress disorder. Additionally, pediatric patients were at a higher risk of cognitive issues.

Interventional Studies

Three interventional studies were included in our review (Table 2). All three studies focused on acute leukemia patients [21-23]. The three studies included a total of 102 acute leukemia patients. Two studies were conducted specifically among children with ALL (combined sample size was 80), while the third included [21] 20 acute leukemia patients without specification. The three studies were conducted among patients receiving chemotherapy (whether induction or maintenance) and were done in Iran. Studies used different psychometric instruments, including the QLQ-C30 (21), Pediatric Quality of Life Inventory (PedsQL) [23], and TNO AZL Children’s Quality of Life - Parents Form (TACQOL-PF) [22].

**Table 2:** Quality of life interventional studies in the Eastern Mediterranean region. ALL = acute lymphocytic leukemia; QoL = quality of life; QLQ-C30 = The European Organization for Research and Treatment of Cancer quality of life; PedsQL = Pediatric Quality of Life Inventory; TACQOL-PF = TNO AZL Children’s Quality of Life - Parents Form

Article	Population and sample size (% of males)	Disease status/Treatment received	Mean age	Methods and aims	Psychometric instruments used	Major findings
Bahrami et al., 2012 [21]	22 adults with acute leukemia patients	Chemotherapy	34.6	Introducing nursing consultation and assessing QoL pre-and post-intervention	QLQ-C30, version 3	There was no significant change in QoL between pre-and post-intervention. Males had lower QoL. Chemotherapy sessions and age were associated with lower QoL
Khodashenas et al., 2017 [23]	20 children with ALL (40)	Chemotherapy	8.8 in the control group, and 10.1 in the research group	10 patients underwent an aerobic exercise program, and 10 patients continued routine treatment	PedsQL	No significant difference in the Quality of Life Index (QLI) between the intervention and control groups. QoL had a significant association with pain and injury and cognitive problems
Hashemi et al., 2011 [22]	60 children with ALL (75)	Diagnosis in the last two years and children on maintenance therapy	8.13 in the control group, and 8.45 in the research group	30 patients’ parents underwent an educational program and 30 patients’ parents did not	TACQOL-PF	Patients of parents who received the educational program had an improvement in their QoL. Patients had an improvement in global QoL and all QoL dimensions after the intervention when compared to before the intervention

All studies had different interventions. For instance, Bahrami et al. [21] investigated the role of nursing consultations in improving the QoL of adult patients with leukemia who were receiving chemotherapy. The intervention consisted of face-to-face group discussions that included nurses, cancer survivors, and at least four newly diagnosed patients. The sessions tackled topics such as cancer, treatments and their side effects, supportive care, and coping mechanisms. The study showed that the intervention did not improve QoL as there was no significant difference in QoL scores before, one week, and one month after the study (F = 0.006, P = 0.99). However, some of the important observations included that males, chemotherapy, and age contributed to lower QoL.

Two studies were conducted among pediatric patients with ALL. Khodashenas et al. [23] investigated the role of aerobic exercise in 20 ALL patients. The patients were distributed into two groups each with 10 patients. The program consisted of an aerobic exercise program, including different activities such as walking and running. Patients had around three hours of training a week (divided into three sessions) for 12 weeks. The study utilized PedsQL and showed no improvement in the QoL index between the two groups; however, parents’ reports showed statistically significant improvement. On the other hand, Hashemi et al. [22] utilized an educational program for the parents of 60 ALL patients divided into two equal groups (intervention and control groups). The intervention included three sessions that discussed topics such as leukemia, treatments, communication, and taking care of patients. The study reported a statistically significant improvement in the QoL scores both globally and in specific dimensions. There was no significant difference in the baseline QoL score between the control and intervention groups in both studies.

Discussion

This systematic review seeks to shed light on the available research performed to evaluate all domains of QoL in hematological malignancies in the EMR. We illustrated that there is a scarcity of studies focusing on patients with hematological malignancies in this region. The studies were limited by number, geographical locations, and disease entities. The majority of the studies were conducted among leukemia patients. Three studies were observational, and three studies were interventional. The sample size of the interventional studies was small (ranging between 20 and 60 patients), two studies used simple randomization, and none were blinded given the nature of the intervention [21,22]. Other than the validation studies, no studies reported MM, chronic leukemia, HL, NHL, and transplant patients (both autologous and allogeneic).

The included studies demonstrated the negative impact of hematological malignancies and therapies on patients’ QoL. The studies showed the association between physical symptoms and QoL in patients, necessitating the importance of addressing these symptoms. However, few studies reported better outcomes in lymphoma patients [9,14]. In addition to physical symptoms, other reported indicators were male gender and chemotherapy, which were shown to lower the HRQoL. The studies showed a decrease in HRQoL in different dimensions, including psychological, mental, physical, and cognitive, which is consistent with the literature from developed countries [4,24-26].

The current literature illustrates an important gap that future studies should tackle. For instance, more studies are needed to establish translated and validated QoL assessment instruments to be applied among patients in the EMR, particularly the most used ones such as the SF-36 and QLQ-C30. There are validated translations for many instruments, but mainly to Arabic and Persian. Table 3 includes some of the major HRQoL tools and the currently available translations according to their websites [27-31]. More HRQoL tools should be translated and validated into more languages including many symptom-specific tools. Additionally, our review showed that the current literature is limited to a few countries, and most of the Eastern Mediterranean countries had no studies investigating QoL in hematologic malignancy patients. More collaboration between different institutes in different countries is needed through regional societies and groups. However, it should be noted that because this study is limited to our search strategy using only Medline databases, full articles, and English-language articles, there are possibly other studies that were not included in this review.

**Table 3:** Examples of HRQoL tools that have been translated to languages spoken in the EMR. HRQoL = Health-related quality of life; EMR = Eastern Mediterranean region

Tool	Description	Translations
General tools		
EuroQoL 5 Dimension 5-Level (EQ-5D)	A generic instrument consisting of five domains, namely, mobility, self-care, usual activities, pain/discomfort, and anxiety/depression Provides a visual analog scale	More than 200 translations, including Arabic, Farsi, Punjabi, and Urdu
36-Item Short Form Survey (SF-36)	Generic, coherent, and easily administered quality-of-life measure Relies on patient self-reporting Monitoring and assessment of care outcomes in adult patients	Dutch, Spanish, Swedish, French, German, Italian, Danish, Japanese, and Arabic
Patient-Reported Outcomes Measurement Information System (PROMIS)	A set of person-centered measures that evaluates and monitors physical, mental, and social health in adults and children	More than 20 translations, including Arabic, Urdu, and Punjabi
Hematology-oncology specific tools		
The European Organization for Research and Treatment of Cancer quality of life (EORTC QLQ-C30)	Widely used tool for assessing the generic aspects of QOL in cancer patients Tumor-specific modules	More than 100 translations, including Arabic and Iranian
The Functional Assessment of Cancer Therapy/General and Site-Specific Subscales (FACIT-G)	Measures four domains of HRQoL in cancer patients, namely, physical, social, emotional, and functional well-being Site-specific modules	More than 70 translations, including Arabic, Farsi, Punjabi, and Urdu

Further, studies are needed in less researched areas and diseases such as lymphoma and MM. Studies should also investigate HRQoL through the different stages of the disease (starting from the time of diagnosis to survivors). None of the included studies discussed the HRQoL of cancer survivors. This should be a focus of future investigations. Studies were included regardless of the publication year as few studies were conducted from this era. Future clinical research should also include patient-reported outcomes reflecting how patients are doing and addressing their general well-being, which impacts patients’ experience [32-34]. The implementation of new technological interventions that help improve QoL, such as physical activity tracking through wearables and the Internet of Things (IoT), might improve our understanding of patient needs [35].

## Conclusions

This systematic review provides insights into the QoL of patients with hematological malignancies in the EMR. The included studies demonstrated the impact of hematological malignancies and treatments on patients’ HRQoL. It also highlights the association between physical symptoms and QoL in cancer patients, emphasizing the importance of addressing these symptoms. The studies were limited by publication year, the number of patients, geographical locations, and disease entities. Future studies in this area are needed to help understand the factors affecting HRQoL in cancer patients in the EMR and ways to improve it.
